# Human Monocyte Subset Distinctions and Function: Insights From Gene Expression Analysis

**DOI:** 10.3389/fimmu.2020.01070

**Published:** 2020-06-04

**Authors:** Sarah Cormican, Matthew D. Griffin

**Affiliations:** ^1^Regenerative Medical Institute (REMEDI) at CÚRAM Centre for Research in Medical Devices, School of Medicine, College of Medicine, Nursing and Health Sciences, National University of Ireland, Galway, Ireland; ^2^Nephrology Services, Galway University Hospitals, Saolta University Health Group, Galway, Ireland

**Keywords:** monocytes, monocyte subsets, inflammation, gene expression, flow cytometry, next generation sequencing, microarray, immune response

## Abstract

Monocytes are a highly plastic innate immune cell population that displays significant heterogeneity within the circulation. Distinct patterns of surface marker expression have become accepted as a basis for distinguishing three monocyte subsets in humans. These phenotypic subsets, termed classical, intermediate and nonclassical, have also been demonstrated to differ in regard to their functional properties and disease associations when studied *in vitro* and *in vivo*. Nonetheless, for the intermediate monocyte subset in particular, functional experiments have yielded conflicting results and some studies point to further levels of heterogeneity. Developments in genetic sequencing technology have provided opportunities to more comprehensively explore the phenotypic and functional differences among conventionally-recognized immune cell subtypes as well as the potential to identify novel subpopulations. In this review, we summarize the transcriptomic evidence in support of the existence of three separate monocyte subsets. We also critically evaluate the insights into subset functional distinctions that have been garnered from monocyte gene expression analysis and the potential utility of such studies to unravel subset-specific functional changes which arise in disease states.

## Introduction

Monocytes are innate immune cells that account for ~10% of nucleated blood cells (1, 2). They play a key role in anti-microbial immunity through direct responses including phagocytosis and cytokine production in addition to regulating other cells of the innate and adaptive immune systems (3). Under inflammatory conditions blood monocytes may transmigrate into tissues and differentiate to replete or supplement tissue-resident mononuclear phagocytic cells (4). Monocytes also play pathophysiological roles in inflammatory diseases. This is best recognized in atherosclerosis, which is increasingly considered to be a chronic inflammatory condition (5). Monocytes adhere to and transmigrate through endothelial cell layers into the vascular intima, where they internalize modified lipid particles to become foam cells (6). Accumulation of these cells, accompanied by production of pro-inflammatory and pro-fibrotic mediators results in atherosclerotic plaque enlargement and/or rupture (7). Given the physiological and pathophysiological importance of monocytes, a greater knowledge of their roles in health and disease has the potential to inform development of therapeutic strategies.

**Figure 1:**
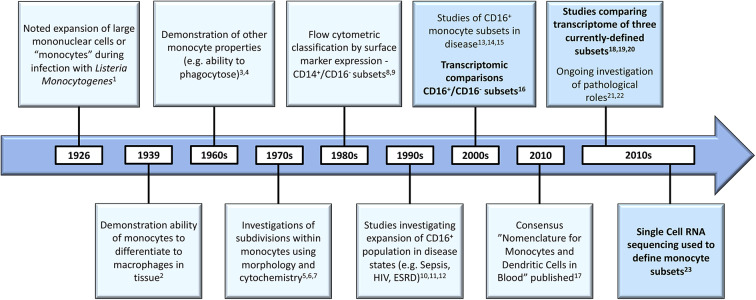
Developments in the understanding of monocyte biology in the past several decades [1. (8), 2. (9); 3. (10); 4. (11); 5. (12); 6. (13); 7. (14); 8. (15); 9. (16); 10. (17); 11. (18); 12. (19); 13. (20); 14. (21); 15. (22); 16. (23); 17. (24); 18. (25); 19. (26); 20. (27); 21. (28); 22. (29); 23. (30)]. Developments in gene expression analysis are bolded and these boxes highlighted. HIV, Human Immunodeficiency Virus; ESRD, End Stage Renal Disease.

**Figure 2:**
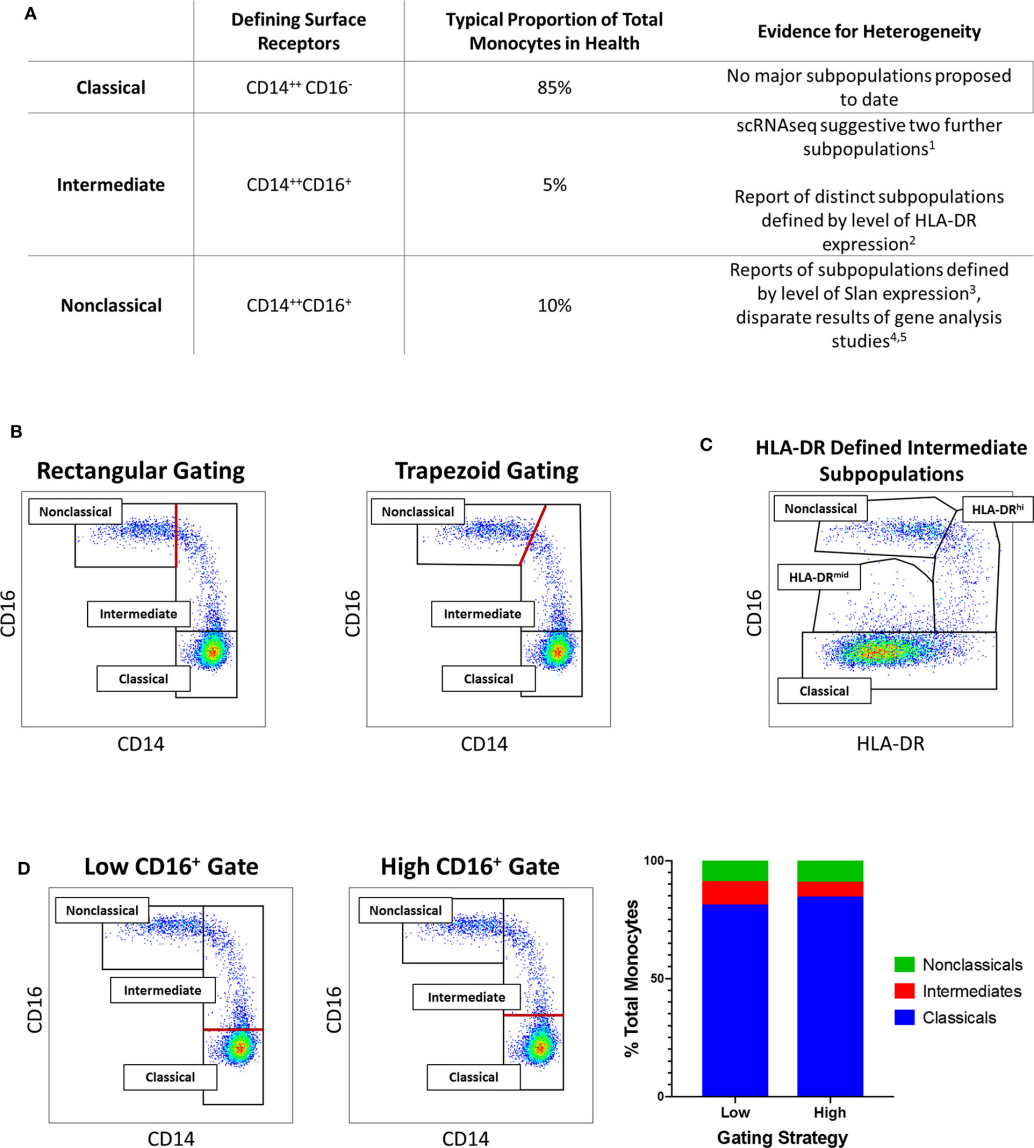
**(A)** Tabular summary of the CD14/CD16 phenotypes, typical proportionate distribution in health and evidence for further heterogeneity of the three currently-recognized monocyte subsets. [1. (30); 2. (31); 3. (32); 4. (25); 5. (33)] **(B)** *Example of variation in distinction of intermediate and nonclassical monocyte subsets*: Flow cytometry dot plots of the three currently-recognized monocyte subsets in peripheral blood mononuclear (PBMC) sample from a healthy adults based on surface expression of CD14 and CD16 [the monocyte population was generated by sequential gating as previously described (29, 31)]. The border between intermediate and nonclassical monocytes may be defined by either a rectangular (left dot plot) or trapezoid (right dot plot) region. **(C)** *Example of intermediate monocyte heterogeneity*: In the same sample, two intermediate monocyte subpopulations in blood from a healthy adult distinguished by mid- and high-level of surface HLA-DR expression as previously described (29, 31). **(D)** *Example of variation in distinction of classical and intermediate monocyte subsets*: In the same sample, setting a low (left dot plot) or high (middle dot plot) threshold for CD16 positivity results in variation in the defined proportions of the classical and intermediate monocyte subset (bar chart, right). In **(B,D)**, the red lines indicate the part of the gating strategy at which variation may occur.

As illustrated in Figure 1, scientific understanding of the physiology of monocytes in health and their properties in disease has progressively increased over several decades (2). A key step along the way was the recognition that monocytes are not simply a homogenous population but are comprised of distinct subsets, which may themselves contain further subpopulations. Development of immunofluorescent flow cytometry in the last decades of the 20th century (15, 16) was an essential technology for the recognition of first two (15) and then three distinct monocyte subsets (Figure 2), defined by relative expression of CD14 (LPS receptor) and CD16 (Fc gamma receptor III) (24). Studies differ, however, as to the exact gating strategy used to separate these three subsets - the separation of intermediate and nonclassical subsets being particularly variable with no consensus as to whether a rhomboid or trapezoid strategy is preferable (illustrated in Figure 2B) (34). Similarly, the distinction between classical and intermediates (based on the cut-off between CD16^−^ and CD16^+^) may vary among individual studies (Figure 2D). Recent work has now confirmed that monocytes egress from the bone marrow as a uniform population of CD14^++^CD16^−^ cells a proportion of which subsequently differentiate to become intermediate and nonclassical monocytes (35). Despite these advances, questions remain regarding the number of monocyte subsets (2) and the pathological roles that individual monocyte subsets play in disease states. With regard to the number of subsets, some authors have argued that subpopulations exist within the intermediate subset (30, 31). In our own work, we have observed that blood intermediate monocytes can be consistently subdivided into subpopulations with high- and mid-level expression of the MHC II protein HLA-DR (Figure 2C) and that the absolute numbers and relative proportions of the two subpopulations are differentially regulated in disease states such as obesity and chronic kidney disease (29, 31). Categorization of CD16^+^ monocyte subsets based on expression of the carbohydrate residue 6-sulfo Lac-NAc (slan) has also been proposed (33). Among the approaches that are available to resolve questions about monocyte phenotypic and functional heterogeneity, gene expression studies may be particularly valuable. It should be noted that a caveat in the interpretation of gene expression studies is that there is an imperfect correlation between mRNA and protein levels due to post-translational modification and other factors (36, 37). Nonetheless, a number of the studies discussed in this review have, to some extent, reinforced their mRNA findings with using additional experimental approaches - an important step in the interpretation of gene expression data.

Between 2006 and 2014, a relatively large number of microarray studies generated gene expression data from total blood monocytes during health or various disease states. The resulting datasets have been used by Rinchai et al. to generate a curated compendium of monocyte transcriptional profiles (38). More recently, however, gene expression analysis techniques have been applied to purified human monocyte subpopulations. The overall impact of these studies on our understanding of monocyte subset biology has not previously been reviewed. In this article, we focus on the knowledge that has been gained from studies that have used a range of gene expression analysis technologies to investigate differences among human monocyte subsets or have sought to determine subset-specific functions and roles in disease.

## Development of Gene Expression Analysis Techniques

Advances in genetic sequencing techniques (summarized in Table 1) have enabled researchers to define immune cell subtypes and to accurately assign functional roles by studying cellular transcriptomic data (43). The “transcriptome” is a quantified measurement of all RNA transcripts present in a cell. Inferences regarding cellular function may be drawn from quantitative and qualitative analysis of gene expression (43). Rapid advances in genetic sequencing technology have reduced costs and increased scalability of transcriptomic studies (47).

**Table 1:** Summary of genomic sequencing technologies utilized by researchers cited in this review.

	**Summary of technique**
Sanger Sequencing (39)	• cDNA amplified using fluorescently labeled nucleic acid, primers, DNA polymerases • Amplified cDNA segments separated in capillary gel • Fluorescent labels used to sequence segments
Microarray (40)	• Microarray chip contains cDNA probes for transcripts of interest • Isolated cDNA hybridized to chip • Microarray chip scanned to quantify gene expression
Tag-based sequencing (SAGE) (41)/Super-SAGE) (42)	• Streptavidin beads used to bind cDNA • Restriction endonuclease cleaves cDNA, cleaved cDNA discarded • Oligonucleotide adaptors added, with binding site for cleaved sticky end (Adaptor contains an upstream tag-enzyme binding-site and a primer site for subsequent steps) • Complementary strand to cDNA produced until cleaved by tagging-enzyme (15 b.p. for standard SAGE) • Final tags annealed to form a contig—multiple DNA “tags” separated by a primer and tag-enzyme binding site • Contig expanded by bacterial replication and sequenced • DNA tags aligned with reference genome
RNA-seq (43)	• cDNA isolated and cleaved into fragments • Fragments sequenced by NGS technologies • Sequences classified [exonic/junctional/Poly(A)] and aligned with reference genome
Massive Analysis of Complementary DNA Ends (44, 45)	• Combination of tag-based approach and NGS • cDNA randomly fragmented • Sequence generated from poly(A) tail on 5′ cDNA strand • Contigs assembled, replicated, sequenced and aligned with reference genome
scRNA-seq (46)	• Single cells sorted into individual wells • Transcriptome for each individual cell generated separately by NGS methods

Traditional Sanger Sequencing was initially used to sequence cellular RNA after conversion to complementary DNA (cDNA) (39). However, this technique is costly, time consuming and does not allow the quantity of each RNA transcript to be determined (43). Microarray analysis was subsequently developed to allow relative quantification of gene expression (40). This technique involves hybridization of cDNA generated from isolated cellular RNA to fluorescently labeled cDNA probes and allows for comparison of gene expression levels among populations of cells. Tag-based RNA sequencing techniques provided a further refinement (41). These approaches [e.g., SAGE (Serial Analysis of Gene Expression) and Super-SAGE] involve production of primers which anneal to the 5' end of RNA transcripts only. A short piece of RNA or “tag” is sequenced for each RNA fragment (15 base pairs for traditional SAGE analysis, longer for super-SAGE) (42) and these tags are then aligned against a reference genome and gene expression levels are inferred based on the frequency of each tag. CAGE (cap analysis of gene expression) is a further variation on tag-based sequencing wherein all tags produced are from transcription start sites (48).

These techniques facilitated studies of cellular gene expression, although some limitations remained (43). For example, microarray data are limited to expression of known genes and “background noise” which occurs due to non-specific probe hybridization requires that data are normalized, making comparisons from different experiments problematic (49). Tag-based sequencing methods overcame these limitations but are costly and the full sequencing information of cellular transcripts remains unknown.

High throughput genetic sequencing methods, also known next generation sequencing (NGS), generate sequences for multiple pieces of genetic material simultaneously (50). These techniques have massively reduced the cost of genetic sequencing and may be applied to extracted RNA to generate quantitative transcriptional data (43). This approach, commonly termed “RNA-seq” has now been applied to many cell types in both healthy and disease states. Identification of differentially expressed genes (DEGs) allows comparisons to be made among different populations of cells or within the same population of cells isolated under different conditions (51). Other recently-developed approaches combine high throughput sequencing with a “tag”-based approach to gene expression analysis. For example, in massive analysis of cDNA ends (MACE), a short stretch of cDNA from the 3' end of each transcript is sequenced. These sequenced tags are mapped to the reference genome and gene expression is thus determined. This approach is cheaper than conventional RNA-seq and may be superior for the identification of rare transcripts (44, 45). Finally, single-cell RNA-sequencing (scRNA-seq) represents a still more recent advance in transcriptomic analysis. Individual cells from within a population are sorted and their RNA expression quantified allowing heterogeneity within cell populations to be determined. This technique may be especially valuable in interrogating subdivisions within immune cell subpopulations (46).

Large amounts of gene expression data require significant bioinformatics expertise to interpret appropriately. It should be emphasized that variations in the bioinformatics analysis techniques employed by the research groups profiling immune cell subpopulations may be as important as differences in the genetic sequencing techniques. An initial step is analysis of DEGs to identify specific individual genes that are highly expressed by a group of cells. Additional analysis of the entire transcriptome, considering the number of DEGs and magnitude of difference, may be used to determine if groups of cells are genetically distinct or to compare the degree of difference among several populations. Multiple studies have compared the degree of similarity among monocyte subsets by considering the total number of DEGs among them (26, 27, 52). Other techniques used to determine if proposed cellular subpopulations are truly distinct include principal component analysis (PCA) and hierarchical clustering. Principal component analysis is a statistical procedure whereby multiple linear, potentially correlated variables (e.g., gene expression magnitude) are converted into non-correlated “components.” This allows data to be visualized in two dimensions and allows for clusters/groups of cells to be identified. For example, as described in detail later, PCA of gene expression data from monocyte subsets from each of several donors has been used to determine that the intermediate monocyte subset is genetically distinct from classical and nonclassical subsets (25). Hierarchical clustering, is an alternative approach used to analyse the relationships among cell subpopulations, with the results usually represented on a dendrogram.

In order to integrate newly-generated data with existing biological knowledge, various approaches have been used to determine the functional implications of gene expression data. Gene set enrichment analysis techniques determine classes of genes which are highly expressed by a cell population. For example, publicly available gene ontology (GO) data may be integrated with DEGs identified to predict biological processes which differ among the populations of cells under comparison (26, 27). Pathway Analysis may be performed, using platforms such as Ingenuity Pathway Analysis (IPA) to integrate newly-generated data with existing knowledge of cause-effect relationships, for example to predict upstream regulators or downstream effects of the observed DEGs (53). Other data mining techniques such as weighted gene co-expression analysis (WGCNA) may be used to identify clusters of co-expressed genes which are up or down regulated (54).

## Gene Expression Analyses of Human Monocytes Subsets in Health (2007–2012)

Transcriptomic evidence for a “monocyte dichotomy” has previously been reviewed (23, 55). Anzbazhagan et al. (56) combined gene expression data from five microarray datasets (57–60) to determine commonly-identified DEGs and identify links between functional properties and transcriptional data. As only one of these papers (26) considered the intermediate monocyte subpopulation separately, this population could not be included in the combined review. It was noted that CD14^+^/CD16^−^ (Classical) monocytes demonstrate high expression of genes involved in responses to bacterial infection and inflammation [e.g., *TLR4* (toll-like receptor 4), *TREM1* (triggering receptor on myeloid cells-1), *CCR2* (chemokine receptor 2)], genes involved in inflammasome signaling [e.g., *NLRP3, NLRP12* (NACHT, LRR and PYD domains-containing proteins 3 &12)] and genes involved in low density lipoprotein (LDL) uptake [e.g., *LDLR* (low density lipoprotein receptor)] (56). In contrast, CD16^+^ monocytes have high expression of genes involved in cytoskeletal dynamics [e.g., *CDC42EP4* (CDC42 effector protein-4), *CKB* (creatinine kinase B), *EML4* (EMAP-like protein 4)], tissue invasion in inflammation [e.g., *CTSL1* (cathepsin 1)] and genes suggesting terminal differentiation and cellular maturity [e.g., *CDKNIC* (cyclin-dependent kinase inhibitor 1C), *HES4* (hairy and enhancer of split 4)] (56). The authors of the papers used by Anbazhagan et al. made their raw data publicly accessible in the Gene Expression Omnibus (GEO) repository allowing a combined approach to data analysis. Other research groups also reported on transcriptional differences between CD14^+^/CD16^−^ and CD16^+^ subsets but these could not be merged for a single analysis. Nonetheless, some common findings were described including higher expression of *CCR2* by the classical subset (55) and cathepsins by the nonclassical subset (55) as well as higher expression of *ITGAM* (the gene for CD11b) by the classical subset (61). In the remainder of this article we will focus on literature investigating the transcriptional profiles of the three currently recognized monocyte subsets.

Within a short time-period following the publication of consensus nomenclature in 2010 (24), three high-quality studies examined genetic distinctions among the three currently recognized monocyte subsets (25–27). Importantly, in these studies (which are summarized and compared in Table 2), the authors attempted to validate the identified differences in gene expression with functional experiments. Cros et al. (25) were the first to purify three monocyte subsets from healthy adults and to compare gene expression by different subsets using a microarray approach. Hierarchical clustering and principal component analysis supported the existing nomenclature as subsets isolated from each donor clustered together. However, in this study, Slan expression did not allow discrimination of genetically-distinct monocyte subpopulations. Of note, the classical and intermediate subsets were the most closely related subsets. Gene expression data for murine monocyte subsets was also generated and, in keeping with previous observations by Ingersoll et al. (58), the human classical and intermediate monocyte subsets were found to most closely resemble mouse Ly6C (Gr1)^hi^ monocytes. Nonclassical monocytes most closely resembled mouse Ly6C^lo^ monocytes, which had recently been reported, in mice, to crawl on the vascular endothelium (62). Given the analogous gene expression profiles of human and mouse monocytes, the authors designed a number of experiments to determine if human monocyte functions aligned with those of their murine counterparts. These experiments confirmed that human nonclassical monocytes patrol the vascular endothelium in similar fashion to mouse Ly6C^lo^ monocytes. Furthermore, classical and intermediate monocytes phagocytosed latex beads and produced reactive oxygen species (ROS) and pro-inflammatory cytokines in a similar manner to mouse Ly6C^hi^ monocytes (25). In summary, this study used gene expression analysis to cluster human and mouse subsets, thus predicting functional roles of human monocyte subsets and these predictions were extensively validated by adoptive transfer and *in vitro* functional experiments.

**Table 2:** Summary of the relevant details of three landmark studies based on gene expression analysis of purified classical, intermediate and nonclassical monocyte subsets in health.

	**Cros et al. (25)**	**Wong et al. (26)**	**Zawada et al. (27)**
Gene expression technique	Microarray	Microarray	SuperSAGE
GEO	E-MEXP-2544 E-MEXP 2545	GSE3081	GSE25931
Most closely related populations	Nonclassical & Intermediate	Classical & Intermediate	Classical & Intermediate
**Demonstrated functional correlations**			
**Subset**	**Cros et al**. **(****25****)**	**Wong et al**. **(****26****)**	**Zawada et al**. **(****27****)**
Classical	Cytokine Production: Highest production IL-8, IL-10, CCL2, CCL3 after LPS stimulation. Also produce IL-6 Phagocytosis of latex beads Produced high levels of ROS	Receptor Expression: Highest expression of CD54, CCR1, CCR2, CXCR1, CXCR2, CXCR4, CD11B, CD33, CD52L, CD1d, CD9, CD99, CLEC4D, CLEC5A, IL13Ra1 Cytokine Production: Produce GM-CSF, IL10, CCL2 after LPS stimulation	Receptor Expression: Highest levels of CD91, CD64, CD11B, CD35 Lowest ROS production Induction of T-cell proliferation
Intermediate	Cytokine Production: Highest production TNF-α, IL-1ß, and IL-6 after LPS stimulation, also produced Phagocytosis of latex beads Did not produce ROS	Receptor Expression: Highest expression of CD40, CD80, HLA-ABC, HLA-DR, CD32, CCR5, CD54, CD163, CLEC10a, GFRa2 Cytokine Production: Intermediate or lowest level production of all cytokines studied	Receptor Expression: Highest levels of CD74, HLA-DR, CD202B, CD105 Highest ROS production Most potent induction of T-cell proliferation *in vitro* Form cellular clusters in an *in vitro* angiogenesis assay
Nonclassical	Cytokine Production: IL-1R antagonist production after LPS stimulation TNF-α and IL-1ß production after viral stimulation Did not phagocytose latex beads Did not produce ROS Patrolling behavior on endothelial layers	Receptor Expression: Highest expression of CXC3CR1, CD115, CD97, CD123, CD 294, P2RX1, Siglec10 Cytokine Production: Highest production of TNF-α and IL-1ß	Receptor Expression: Highest levels of CD31, CD43, CD11a, CD47 Intermediate ROS production Induction of T- cell Proliferation *in-vitro*

Two other studies characterized gene expression profiles of the three currently accepted monocyte subsets. In contrast to the study by Cros et al., however, the results reported from these studies favored a conclusion that intermediate monocytes are more closely related to nonclassical than classical monocytes (26, 27). Zawada et al. used SuperSAGE analysis to identify DEGs among the three monocyte subsets (27). By this analysis, intermediate and nonclassical subsets were deemed most closely related with 559 DEGs compared to 1127 DEGs between classical and intermediate subsets. Gene ontological studies were then used to predict biological processes enhanced in each subset. *In vitro* experiments relevant to identified processes were then performed, elegantly demonstrating the power of gene expression studies to determine cellular function. For example, intermediate monocytes were shown to have high expression of MHC-II-restricted antigen processing and presentation related genes and it was then demonstrated that these cells are capable of stimulating T-cell proliferation (27). Expression of genes related to cellular processes including cell adhesion, oxidative stress and phagocytosis differed across subsets (27). Production of ROS by unstimulated monocyte subsets showed a high level of basal production in intermediate monocytes, mirroring this subset's high expression of genes involved in superoxide regulation (27). Across subsets, cell surface marker expression levels (measured by flow cytometry) correlated with expression levels of the related genes (27). The third large study of gene expression profiling of monocyte subsets by Wong et al. used microarray technology and again reported that intermediate monocytes are most closely related to nonclassical monocytes (26). These authors also used gene ontology analysis to infer biological processes performed by each subset. Of note, genes involved in cell movement were highly expressed by the nonclassical subset, in keeping with their reported patrolling behavior. Similar to Zawada et al., genes involved in MHC-II processing and presentation were found to be highly expressed by the intermediate subset and genes for S100 proteins were highly expressed by the classical subset (26).

Taken together, these studies demonstrated that gene expression analysis is a powerful tool to predict cellular function. The importance of *in vitro* experiments to validate functional predictions based on gene expression data was also illustrated. Nonetheless, a number of gaps and inconsistencies remain in fully understanding subset-specific functions. These relate in particular to the intermediate monocyte subset which is variously described as most closely resembling either the classical or nonclassical subset. Other physiological roles including inflammatory cytokine production and angiogenic capability have also been assigned to different subsets by different authors. As described in the Introduction and illustrated in Figures 2B–D, one issue which may lead to discrepancies among studies is that the definition of flow cytometry analysis and sorting gates is not uniformly standardized protocol. The use of different methodologies, including microarray and tag-based approaches, to analyse gene expression is also likely to explain some of the variation within published literature.

## More Recent Gene Expression Analyses of Human Monocytes Subsets in Health

In an innovative study, Schmidl et al. investigated differential gene expression among monocyte subsets (48) and coupled these profiles with analysis of mechanisms of gene expression regulation. The authors initially confirmed that the three recognized monocyte subsets clustered separately on multidimensional scaling analysis. A total of 10,249 protein-encoding genes was identified with a higher number of DEGs between the intermediate and classical monocyte subsets than between the intermediate and nonclassical. Chromatin immunoprecipitation sequencing (ChIP-seq) analysis was then used to identify subset-specific histone modifications (H3K4me1 and H2K27ac) in the classical and nonclassical subsets. Subset-specific differential CAGE cluster expression was analyzed in all subsets and was found to correlate with histone modifications.

The authors next sought to identify differentially utilized transcriptional motifs by performing *de novo* motif detection in regions of subset-specific histone modifications or CAGE cluster expression. The classical monocyte motif signature was dominated by AP-1 (activator protein 1) and CEBP (CCAAT enhancer binder protein), the intermediate monocyte subset by NF-κB (nuclear factor-κB), E-box and MEF2 (myocyte enhancer factor 2) motifs while the nonclassical signature included E2F, NRF1 (nuclear respiratory factor 1) and OCT (octamer transcription factor) motifs. Some of these motifs corresponded to differential expression of the mRNA for specific transcription factors. For example, classical monocytes exhibited higher expression of the FOS transcription factor, a component of AP-1. Finally, gene ontology analysis yielded findings consistent with previous work—for example, upregulation of genes associated with antigen processing and presentation in intermediate monocytes. This analysis also suggested that classical and nonclassical monocytes differ in metabolic processes with upregulation of glycolytic pathways in classical monocytes and oxidative phosphorylation pathways in nonclassical monocytes. In addition to confirming that the three monocyte subsets are genetically distinct, this study pointed to epigenetic regulatory mechanisms underpinning differential gene expression.

In 2015, Hofer et al. re-examined the use of slan to discriminate monocyte subsets (33). The authors proposed that this glycan marker be used to more clearly differentiate the intermediate and nonclassical subsets - an approach that could potentially overcome difficulty in standardizing gating of monocyte subsets. Firstly, a conventional CD14/CD16 based gating strategy was contrasted with a strategy which distinguished subsets of CD16^+^ monocytes on the basis of slan positivity (with slan^+^ cells corresponding to the conventional nonclassical subset). Next, MACE analysis was conducted on monocyte subsets sorted using magnetic column-based isolation. An observation that MHC-II genes related to antigen presentation were highly expressed by the intermediate subset was common to both isolation strategies and consistent with Zawada et al. (27). In this study, PCA supported the separation of conventionally-defined intermediate and nonclassical monocytes as well as separation of CD16^+^ monocytes on the basis of slan expression [an observation that diverges from Cros et al. (25)]. It should be noted, however, that while intermediate monocytes were reliably CD16^+^Slan^−^, some cells which would have fallen within the conventional nonclassical gate were also Slan^−^. This study identified DEGs between intermediate and nonclassical monocytes using a rectangular CD14-based gating strategy and DEGs between Slan^+^ and Slan^−^ CD16^+^ monocytes. More DEGs (676) were identified using a CD14 based separation than a Slan based separation (385) and 314 genes were common to both approaches. A cluster of genes related to antigen presentation was identified on interaction analysis (33). The dominant GO term discriminating Slan^+^ and Slan^−^ monocytes was “regulation of cytokine production.” Interaction analysis demonstrated that the Slan^+^ monocytes highly expressed a cluster of genes related to Ubiquitin C, which functions in the regulation of diverse cellular processes.

In 2017, Metcalf et al. compared gene expression profiles of FACS-purified monocyte subsets from young and older individuals, using microarray analysis (63). Hierarchical clustering was employed to examine the aggregation of samples on the basis of monocyte subset and age of the individuals. While the three previously-described subsets aggregated together, this analysis did not discriminate young and older subjects (for any subset). In this study, classical and intermediate monocytes were the most closely related subsets on hierarchical clustering analysis. Further analyses of unstimulated monocyte subsets were performed with combined data for all subjects. The authors first considered differences between CD16^−^ and CD16^+^ monocytes and reported higher expression of transcripts including *TNF* (tumor necrosis factor), *CX3CR1* (fractalkine receptor) and *IFNG* (interferon gamma) for CD16^+^ monocytes and higher expression of transcripts including *SELL2* (L-selectin), *CCR1/2* (CC-chemokine receptors 1 and 2) and *TLR2/4/5/6/8* (toll like receptors 2/4/5/6/8) in CD16^−^ monocytes. Other observations included that some genes for MHC-II molecules were highly expressed by both classical and intermediate monocytes while others were more highly expressed by intermediates only and that transcripts related to cytoskeletal organization were more highly expressed by nonclassicals. These findings were broadly consistent with previous work (25–27). Next, the authors purified monocyte subsets and stimulated them *ex vivo* with pathogen recognition receptor (PPR) agonists [LPS, a TLR4 agonist; CLO97, a TLR7/8 agonist and 5pppRNA, a RIG-1 (Retinoic-acid inducible gene I) agonist]. After stimulation, each subset retained a distinct gene expression profile, although the number of DEGs was increased. Gene expression after stimulation was compared with controls to determine the effect of stimulation on transcriptomic activity. Taking the studied subjects as a whole, agonist stimulation resulted in a number of differences to unstimulated cells. For example, 5'pppRNA-treated classical and intermediate monocytes upregulated expression of interferon-related transcripts. Some differences in the transcriptional response of monocyte subsets from young and older subjects were also observed. Most notably, classical monocytes from older subjects did not upregulate interferon-related transcripts to the same extent as those from young subjects after stimulation with 5'pppRNA. This observation was further validated by demonstrating lower IFN-α levels in the supernatants of 5'pppRNA-stimulated classical monocytes from old subjects (63). 5'pppRNA-stimulated classical monocytes from young adults also had a greater enrichment of transcripts for costimulatory molecules including CD80 and some cytokines including IL-15 and CCL19.

Differential expression of microRNAs among the three currently recognized monocyte subsets has also been reported (64). In this analysis, the intermediate and nonclassical subsets were most closely related in terms of miRNA profile. Focussing on intermediate monocytes, this subset differentially expressed 38 miRNAs known to regulate biological processes including TLR- and cytokine-mediated signaling, phagocytosis, antigen presentation and processing and lipid/triglyceride metabolism. Relative to the other subsets, the most highly-expressed miRNA was miR-6087 and the lowest relative expression was observed for miR-150p. The functional roles of these specific miRNAs are not fully understood although mi-R150p may regulate inflammatory responses (65) and miR-6087 (66) may contribute to angiogenic potential. Other authors have studied differential expression of miRNA by the classical and nonclassical subsets but without including intermediate monocytes in their analysis (67).

As discussed above, single-cell analysis of gene expression may be a powerful tool to identify previously unrecognized subpopulations and determine function. For example, Gren et al. (68) performed gene expression analysis after single cell sorting of human monocytes. In this work, PCR of 85 genes, rather than analysis of the whole transcriptome, was used to compare subsets (68). While clustering of the three recognized subsets was observed on principal component analysis, the authors also highlighted that significant intercellular variation existed within the subsets.

In an extensive 2017 study, Villani et al. used scRNA-seq and unbiased genetic analysis to identify subpopulations of monocytes and dendritic cells from healthy human blood (30). Clusters of cells with similar gene expression patterns were identified using t-distributed stochastic neighbor embedding (t-SNE), a machine learning approach that allows visualization of complex data within a two-dimensional space (69). Using this approach, the conventionally-defined classical and nonclassical subpopulations were largely contained within two major transcriptionally-defined clusters (termed Mono1 and Mono2) that were separate from each other and all dendritic cell populations. Interestingly, more than half of the conventionally-defined intermediate monocytes were also contained within these two major genetic clusters—predominantly co-clustering with classical monocytes. This observation suggests that a high proportion of intermediate monocytes may not be fully distinguishable from classical or non-classical monocytes at a transcriptional level and fits with the fact that variations in gating approaches may over- or under-estimate the frequency of this subset. The remainder of the conventionally-defined intermediate monocytes, along with a smaller proportion of the non-classical monocytes, formed two additional genetically-defined clusters which expressed distinct transcriptional signatures linked to cell cycle, differentiation and trafficking (termed Mono3) and cytotoxicity (termed Mono4), respectively (30).

While these results suggested that intermediate monocytes, as currently defined, may consist of multiple known and previously unknown subpopulations, the existence of new monocyte subtypes within the intermediate gate has since been called into question (70, 71). In 2019, Zillionis et al. reported results from an innovative scRNA-seq analysis of myeloid cells in the peripheral blood as well as in the tumor tissue of seven individuals with non-small cell lung cancer (NSCLC) (70). In this study, three genetically-defined monocyte subsets (Mono_1−3_) were identified in both blood and tumor tissue of the human subjects as well as in the tumor tissue of a mouse model. In both humans and mice, Mono_1_ and Mono_2_ correlated with the classical and nonclassical monocyte subsets and also closely matched the genetically-defined Mono1 and Mono2 subtypes reported by Villani et al. (30). Furthermore, in both species, the third monocyte population identified by Zilionis et al. (70), Mono_3_, corresponded to the (predominantly intermediate) Mono3 cluster from the study of Villani et al. (30) and was characterized by expression of a set of neutrophil-associated genes. In other respects, however, the genetic profile of Mono_3_ in humans overlapped with that of CD14^+^/CD16^−^ monocytes and was suggested by Zilionis et al. (70) to represent a subpopulation of classical monocytes. The Mono4 population reported by Villani et al. (30) was not identified by Zilionis et al. (70) and the authors proposed that this gene signature may have been derived from physical doublets with NK cells—a conclusion that was also reached by Günther and Schultze (71). Although the studies of Villani et al. and Zillionis et al. differed in that they analyzed cells from blood samples of healthy volunteers and cancer patients respectively, (30, 70), it, nonetheless, remains questionable whether one or more genetically-distinct intermediate monocyte subsets can be fully distinguished from classical- and non-classical-type monocytes in single cell analyses.

To summarize, gene expression analyses since the publication of a consensus nomenclature for monocyte subsets have broadly validated the existence of three genetically distinct monocyte subsets. However, the reported similarity between subsets varies, most notably with a lack of consensus on whether the intermediate monocytes most closely resemble the classical or nonclassical subsets. One explanation for the discrepancies in regard to intermediate monocytes may lie in the observations of Villani et al. and others which indicate that heterogeneity exists within this subset (29–31, 72). Alternatively, cells defined by flow cytometry as intermediate monocytes may represent, at least in part, a mix of classical and non-classical monocytes transitioning between various states of activation or differentiation without a stable, singular identity. We propose, therefore, that further attention to heterogeneity within the intermediate subset will be necessary to fully resolve the relationships among the three currently recognized monocyte subsets.

## Gene Expression Analyses of Monocyte Subsets During Disease States

Changes in immune cell activity occur in disease states, either as a protective mechanism or as part of the disease pathogenesis. Alterations to the circulating monocyte subset profile occur commonly in acute and chronic disease settings. In particular, absolute or proportional increases in the intermediate and/or nonclassical monocyte subsets have been repeatedly reported in diverse conditions including sepsis, HIV, chronic kidney disease, inflammatory bowel disease, diabetes, obesity and atherosclerotic cardiovascular disease (28, 73). Whether individual subpopulations also adopt different functional activities in disease states is less well understood and gene expression analysis techniques may be especially valuable for addressing such questions.

In this regard, some studies have investigated alterations in gene expression by total blood monocytes in disease states. The reader is referred to Rinchai et al. for a complete summary of this literature, a selection of which is discussed briefly here (38). For example, in HIV, Rempel et al. demonstrated that monocytes from patients with uncontrolled infection have an activated phenotype with high expression of genes relating to immune activation and response to stress (74). In the recent study of Dobbs et al., monocytes from children with acute malaria were found to have a different gene expression signature to matching samples obtained after recovery. This signature consisted of 125 DEGs including higher expression of genes encoding complement components and proteins related to TLR signaling during infection (75). Changes in monocyte gene expression in non-infectious conditions have also been investigated. In a study by Liu et al., gene expression in monocytes was correlated with atherosclerotic plaque severity (determined by coronary artery calcium (CAC) score on computed tomography or carotid plaque thickness on carotid ultrasound scan) (76). Expression levels of 13 genes were associated with CAC score and 2 with carotid plaque thickness. Expression levels of two genes were associated with atherosclerosis at both sites: *ARID5B* (AT-rich interactive domain-containing protein 5B), a transcriptional co-activator involved in metabolic activities such as adipogenesis and *PDLIM7* (PDZ and LIM domain protein 7) which promotes mineralization (76). The authors used WGCNA to identify co-expressed gene network modules, three of which were associated with CAC score. Notably, one of these was a cholesterol metabolism transcriptional network including upregulated genes such as *LDLR* (low density lipoprotein receptor) and downregulated genes such as *ABCG1* (ATP-binding cassette sub-family G member 1) expected to result in increased intracellular cholesterol levels. Next, the association of DNA-methylation in monocytes with atherosclerosis was investigated. In this analysis, DNA methylation levels at 31 and 7 sites, respectively, were associated with CAC score and carotid plaque thickness. Most notably, hypomethylation of one CpG site within the *ARID5B* intron (cg25953130) in monocytes was associated with higher CAC scores and also with *ARID5B* mRNA levels. Further *in-vivo* functional analyses demonstrated that *ARID5B* knockdown in a human monocytic cell line (THP-1) results in altered expression of 2,482 other genes, in reduced *IL1A* and IL-1α protein production after LPS stimulation, in reduced migration toward a CCL2 gradient and in reduced phagocytosis. The integration of epigenomic and transcriptomic data in this study provides an elegant example of the use of genetic analyses to identify and validate disease-associated functional pathways in purified monocytes from human patients.

Importantly, however, the studies described above considered monocytes as a single population and studies of subset-specific changes in monocyte gene expression during disease states remain infrequent. An example of the potential can be seen in the previously-described study by Metcalf et al. of the effect of aging on monocyte subset gene expression profiles (63). Monocytes from older subjects expressed higher levels of the chemokine receptor CX3CR1 (63), which mediates trafficking of CD16^+^ monocytes into tissue (77) and could, thus, contribute to age related atherosclerosis. Metcalf et al. also observed a weaker response of classical monocytes from older subjects to viral agonists, both in terms of mRNA expression and functional assays (63). These observations were proposed to underly greater vulnerability to influenza in older adults. Of relevance to current events, high mortality rates have been seen in the elderly during the current outbreak of coronavirus disease (COVID-19), which is caused by severe acute respiratory syndrome coronavirus 2 (SARS-CoV-2) (78, 79). The relatively weaker response of monocytes from older adults to viral agonists reported by Metcalf et al. (63) could explain this observation although further work would be needed to determine if it is a contributing factor to mortality during the pandemic. Although Zillionis et al. used samples from people with non-small cell lung cancer to perform sc-RNAseq a comparison with healthy volunteers was not performed (70).

Still more recent work by Ruiz-Limon et al. (80) determined monocyte subset gene expression profiles in people with rheumatoid arthritis (RA), compared to healthy controls. In this study, CD14^+^ and CD16^+^ monocytes were sorted using magnetic beads (hereafter termed “CD14^+^ monocytes” and “CD16^+^ monocytes”). Gene expression in these monocyte subsets was then compared by PCR array of 84 genes related to atherosclerosis and miRNA expression was compared using a nanostring miRNA array. Although this sorting strategy does not provide full separation of the three monocyte subsets, it did allow genetic changes occurring in CD16^+^ monocytes to be considered separately. Interestingly, in CD16^+^ monocytes isolated from RA patients, 14 genes related to atherosclerosis were expressed at higher levels and 7 at lower levels than CD16^+^ monocytes from healthy controls. These included genes related to inflammation [e.g., *IFN*γ (interferon-gamma) and *CCL2* (chemokine ligand 2)] and lipid metabolism *LDLR (low-density-lipoprotein-receptor)]*. A greater number of miRNAs had altered expression in CD16^+^ monocytes, compared to CD14^+^ monocytes and Ingenuity pathway analysis linked a number of these miRNAs with atherosclerosis-related genes. Significantly, the levels of a number of highly-expressed mRNAs in both CD14^+^ and CD16^+^ monocytes of RA patients correlated with atherosclerosis severity, as assessed by carotid intima media thickness ratio (74). Overall, the study provided evidence that the known increase in cardiovascular disease associated with RA may be promoted by activation of distinctive pro-inflammatory and pro-atherogenic pathways in multiple monocyte subsets. Finally, a further relevant example comes from the recent work of Nowlin et al. who utilized a non-human primate model to determine the effect of acquired immunodeficiency syndrome (AIDS) on monocyte subset-specific gene expression (52). In Rhesus macaques infected with simian immune deficiency virus (SIV), the authors observed that intermediate monocytes more closely resembled nonclassical monocytes as SIV progressed. Based on identified changes in genes relating to the adaptive immune response, it was hypothesized that SIV infection impairs monocyte ability to regulate T-lymphocytes.

Practical considerations may have contributed to the limited use of monocyte subset gene expression analysis in patient cohorts. A relatively large volume of blood is needed to purify sufficient numbers of infrequent blood leukocyte populations to allow for RNA sequencing and this may not be practical in some clinical settings. Purification of subsets from individuals may also be complicated by changes in surface marker expression which have been reported in disease states (81) and which could affect standard gating approaches. Although gene expression technologies such as RNAseq are becoming cheaper and now require smaller amounts of RNA to generate sequencing data (47), there remain significant cost implications in generating gene expression data from multiple purified subpopulations from each patient and control subject within a study cohort. Acquisition and complex processing of samples from clinical settings over prolonged periods of time also pose important logistical and data analysis challenges. Cryopreservation of cells and subsequent sorting, as performed by Dobbs et al., may be a valuable approach to facilitate longitudinal studies (75), although differential effects of cryopreservation on monocyte subset viability and surface markers must be carefully excluded. Finally, we emphasize that careful matching of control subjects (for age and co-morbidity status) is crucial to avoid confounding in such studies.

## Conclusion and Future Directions

Studies of gene expression in the last decade have confirmed the existence of at least three distinct monocyte subsets in humans and have increased scientific understanding of the physiological role of each subset. The landmark studies of Cros et al. (25), Wong et al. (26), and Zawada et al. (27) are notable for the combined use of gene expression analysis and complementary *in vitro* functional studies of purified monocytes subsets. A more recent application of scRNA-seq raised the possibility that the intermediate monocyte subset may contain classical- and non-classical-like monocytes as well other distinct monocyte subtypes (30). However, the latter observation was not corroborated by a subsequent single cell transcriptional study which suggested that a novel fourth monocyte subpopulation reported by Villani et al. is likely to have been artifactual and not relevant to understanding intermediate monocytes (70, 71). The work of Metcalf et al. and Ruiz-Limon et al. has demonstrated that gene expression analysis of each subset may be used to compare monocyte phenotype and function between two groups (63). Further carefully-planned use of these techniques could greatly impact on current understanding of the functional heterogeneity and plasticity of each monocyte subset as well as their individual protective and pathogenic roles in many disease states. Monocyte numbers have been shown to be modified by therapeutic immunosuppression in autoimmune diseases such as inflammatory bowel disease (82) and more longitudinal studies of monocyte subset transcriptomic responses to such therapies will be of significant value.

**Figure 3:**
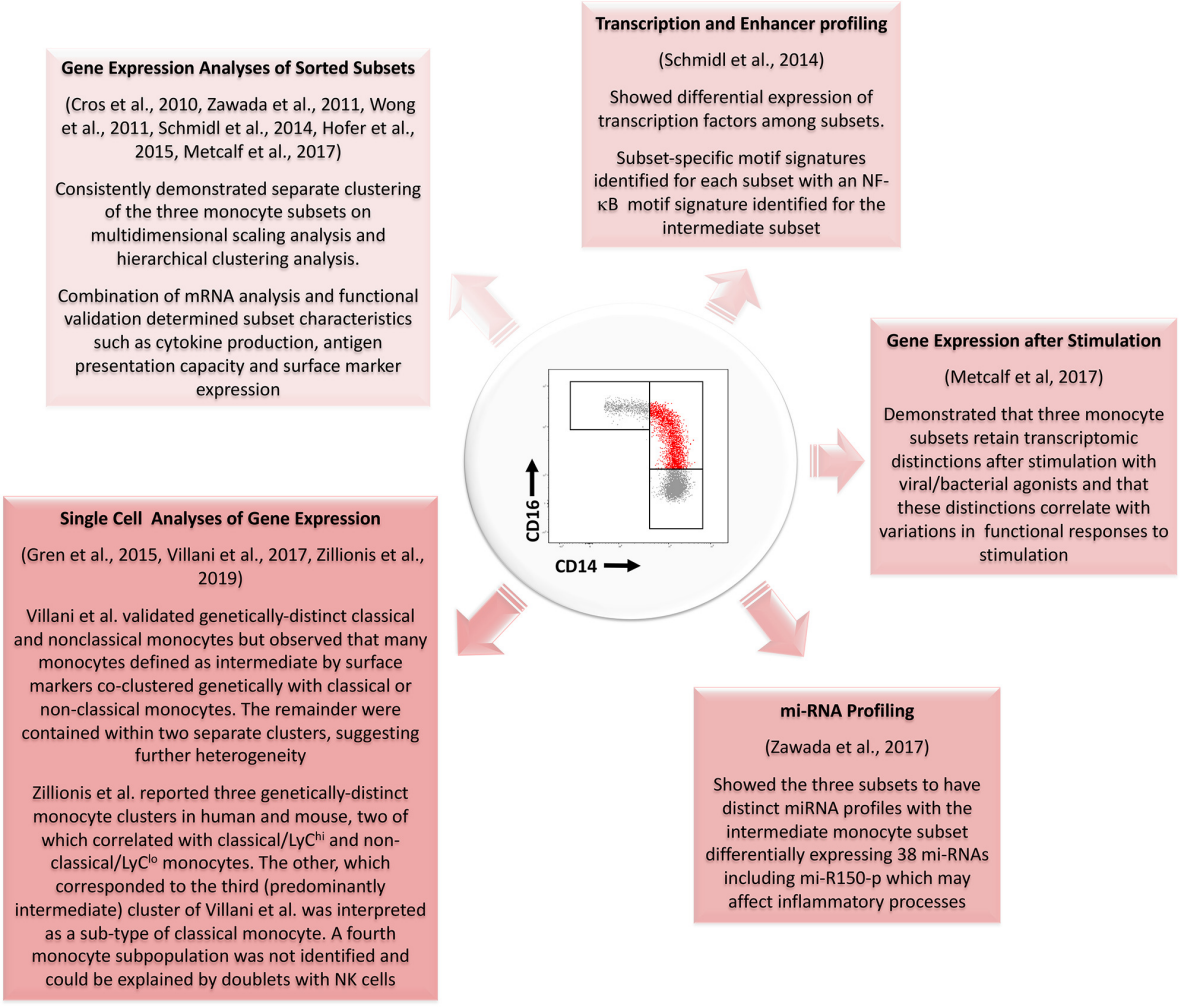
Summary of evidence from different genetic profiling strategies for the existence of a genetically distinct intermediate monocyte subset with reference to studies performed since the development of consensus nomenclature in 2010. Several studies using gene expression analyses of sorted populations have confirmed genetic distinctions between the three populations. Profiling of transcription and enhancer activity and of miRNA profiles has further added to these data. Single cell analyses of gene expression have also generally supported the existence of three monocyte subsets although results from Zillionis et al. (70) suggest the third identified population may represent a subpopulation of classical monocytes. The three monocyte subsets also remain genetically distinct after stimulation with bacterial or viral agonists. mRNA, messenger RNA; NF-κB, Nuclear Factor κB; mi-RNA, micro-RNA.

It is of interest that, despite the quality of the published studies described here, no clear consensus has been reached on whether intermediate monocytes more closely resemble the classical or nonclassical subsets. While variations in the purification strategies for intermediate monocytes may explain some of the differences among reported studies, evidence is accumulating of other forms of heterogeneity within this monocyte subset during health (29–31, 72). Figure 3 summarizes key evidence from profiling studies that monocytes which we currently define as intermediate based on a limited number of surface markers constitute one or more distinctive subpopulations at a genetic/molecular level. It remains unclear, however, whether intermediate monocytes can be unequivocally resolved into a stable functional phenotype at the level of single cell transcriptomes as they appear to have substantial overlap with both classical and non-classical gene expression signatures. Wider adaptation and collaborative analysis of single cell gene expression analyses by the monocyte research community may be the key to resolving such unanswered questions (71). More importantly, it may provide a powerful means to reveal and exploit the dynamic roles of monocyte subsets in the pathogenesis and prognosis of common, life-limiting diseases.

## Author Contributions

SC wrote the first draft of the manuscript and revised subsequent drafts. MG reviewed and revised the manuscript.

## Conflict of Interest

The authors declare that the research was conducted in the absence of any commercial or financial relationships that could be construed as a potential conflict of interest.
